# Perivascular adipocyte size is related to the lipid profile and inflammatory changes in a healthy population

**DOI:** 10.1080/21623945.2025.2499500

**Published:** 2025-05-23

**Authors:** Hana Bartuskova, Ivana Kralova Lesna, Sona Kauerova, Vera Lanska, Jiri Fronek, Libor Janousek, Barbora Muffova, Karel Paukner, Rudolf Poledne

**Affiliations:** a Atherosclerosis Research Laboratory, Experimental Medicine Center, Institute for Clinical and Experimental Medicine, Prague, Czech Republic; b Department of Physiology, Faculty of Science, Charles University, Prague, Czech Republic; c Department of Anaesthesiology, Resuscitation and Intensive Care Medicine, 1st Faculty of Medicine of Charles University and Military University Hospital, Prague, Czech Republic; d Department of Data Science and Statistics, Information Technology Division, Institute for Clinical and Experimental Medicine, Prague, Czech Republic; e Transplantation Surgery Department, Transplantation Center, Institute for Clinical and Experimental Medicine, Prague, Czech Republic

**Keywords:** Perivascular adipose tissue, macrophages, adipocyte size, cardiovascular risk factors, inflammation

## Abstract

Inflammatory changes in perivascular adipose tissue are associated with atherosclerotic lesions in the adjacent artery and can also be used as a marker in patient workup. While adipocyte size is known to be closely related to adipose tissue dysfunction and inflammation, it has not been widely studied in perivascular adipose tissue obtained from healthy human subjects without clinical atherosclerosis. In this cross-sectional study, we addressed this issue by measuring adipocyte size and defining its relationship to cardiovascular risk factors in a healthy cohort of living kidney donors. The presence of cardiovascular risk factors was established by a standardized questionnaire, clinical measurements and body composition analyses. Adipocyte size was measured in the perivascular depot. The proportions of various macrophage subtypes were determined by flow cytometry. To confirm the results, the proportion of CD68  +  macrophages was additionally assessed by immunohistochemistry. A correlation and principal component analyses were performed to explore associations. Adipocyte size in perivascular adipose tissue correlated with markers of lipid metabolism, inflammation, and glucose metabolism. Further, the positive correlation with the pro-inflammatory subpopulation of macrophages suggests a strong local effect of perivascular adipose tissue. Perivascular adipocyte size was associated with cardiovascular risk factors and markers of inflammation in a healthy cohort of living kidney donors. This further supports the local role of adipose tissue dysfunction and inflammation in early atherosclerosis development and detection.

## GRAPHICAL ABSTRACT

**Figure:**
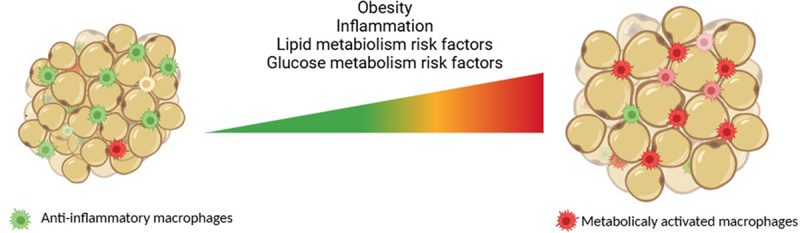
The graphical abstract was created with BioRender. The sentence “Created with BioRender.com” should be put somewhere in the text (e. g. in small letters under the image). However, it is not necessary to add this directly in the Abstract. The sentence can be anywhere in the text, for example in the Methods section. Alternatively, we can add the sentence directly to the image if you think it would be a better solution.

## 1. Introduction

Obesity has become known to be an independent risk factor for insulin resistance (IR), type-2 diabetes mellitus [1], and cardiovascular disease [2]. Adipose tissue expansion occurs through adipocyte hypertrophy and hyperplasia. In the early stage of hypertrophy, lipid droplet size and oxidative stress in adipocytes increase substantially [3]. Adipocyte hypertrophy is also closely associated with lysosomal permeability, subsequent mitochondrial dysfunction, and increased reactive oxygen species production [3,4]. The ability of larger adipocytes to respond to insulin stimulation and store fatty acids is lower compared with that of smaller adipocytes [5,6]. Likewise, cytokine production differs between large and small adipocytes. Specifically, the production and secretion of leptin, IL-6, IL-8, and MCP-1 are increased in large adipocytes [7]. Adipocyte size changes substantially after overfeeding or dietary weight loss, and is tightly associated with changes in whole-body insulin sensitivity, lipid parameters, and metabolism [8,9], as documented, e.g. by enlarged epicardial adipocytes in patients with coronary artery disease [10]. Adipocyte size can be therefore used as a marker of adipose tissue dysfunction and a predictor of cardio-metabolic alterations in overweight and obese individuals [11].

Adipocyte hypertrophy is also closely associated with adipocyte death together with increased infiltration of macrophages into adipose tissue [4,12,13]. Macrophages play an important role in adipose tissue inflammation in obesity [14]; they are partly responsible for the upregulation of pro-inflammatory cytokines such as TNF-α and IL-6 in obese adipose tissue [14,15]. Pro-inflammatory cytokines produced in adipose tissue can act in both local and systemic manners [16]. We have previously repeatedly documented that the proportion of resident metabolically active pro-inflammatory adipose tissue macrophages (MAP-ATMs) is associated with cardiovascular disease risk factors such as hypercholesterolemia, age, and male sex in the adipose tissue of healthy humans [17,18].

Perivascular adipose tissue (PVAT) directly influences the vessel wall [19]. To illustrate this point, the adiponectin produced in PVAT facilitates vascular relaxation through nitric oxide (NO) bioavailability. This effect is attenuated in subjects with metabolic syndrome; possibly as a direct consequence of inflammation and hypoxia in obese adipose tissue [20]. Polarization of macrophages in PVAT has been shown to be closely related to the risk properties of the coronary plaque [21]. Macrophage activation in adipose tissue is also involved in the loss of PVAT’s anti-contractile effect upon acute stimulus in an experimental model [22]. Conversely, endovascular injury leads to rapid pro-inflammatory changes mediated by TNF-α in the adjacent adipose tissue [23]. Changes in adipocyte morphology induced by PVAT inflammation can be detected non-invasively by computed tomography and could serve as a marker of vascular inflammation with a possible detection of unstable lesions [24]. This further highlights the close bi-directional relationship between the vessel wall and adjacent PVAT. However, the extent to which adipose tissue inflammation can influence the arterial wall remains yet to be elucidated. Moreover, data regarding perivascular adipocyte size in healthy populations are scarce.

Given the above, we examined adipocyte size and macrophage burden in PVAT in a relatively healthy cohort of living kidney donors (LKDs) and studied its relationship to the presence of cardiovascular risk factors and markers of inflammation.

## 2. Results

To document that the LKD cohort represented a healthy population, the donors were compared to sex and age-matched individuals selected from the latest Czech post-MONICA study [25] investigating 1% population sample from several districts. As shown in the Table 1, body mass index (BMI) and lipid profile were comparable or healthier in the living kidney donor group with the exception of triglyceride (TG) levels.

**Table 1.** Clinical characteristics.

	LKDs (*N* = 69)	MONICA (*N* = 69)	p
Age (years)	52.44 ± 11.03	52.23 ± 9.03	NS
Male (n; %)	21; 30.43	21; 30.43	NS
Total cholesterol (mmol/l)	4.56 ± 0.90	5.36 ± 0.91	<0.0001
LDL-C (mmol/l)	2.86 ± 0.83	3.18 ± 0.80	0.0193
HDL-C (mmol/l)	1.34 ± 0.42	1.62 ± 0.45	0.0003
TG (mmol/l)	1.65 ± 0.84	1.23 ± 0.60	0.0002
BMI (kg/m^2^)	26.08 ± 3.49	27.81 ± 5.18	0.0231
Waist circumference (cm)	91.59 ± 10.63	–	–
Waist-to-hip ratio	0.92 ± 0.09	–	–
Body fat (%)	29.44 ± 7.60	–	–
Statin use (n; %)	6; 8.70	–	–
hs-CRP (mg/l)*	1.15 ± 1.06	–	–

Average ± SD.

*n = 61.

### 2.1. Adipocyte size and its relationship to the presence of cardiovascular risk factors

**Figure 1.**
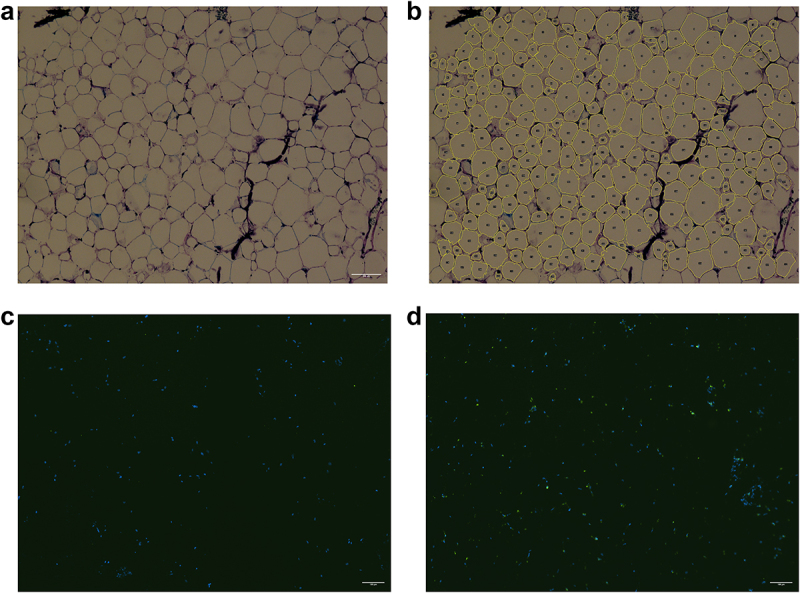
Representative images from histological and immunohistochemical analysis. PVAT section used for adipocyte size analysis – original image (a) and post-analysis (b), magnification 10×; PVAT section used for immunohistochemical analysis – isotype (c) and positive CD68 staining (d); magnification 10×; nuclei in blue (DAPI), CD68 in green (DyLight 488); contrast and brightness were changed in a linear manner (identically for both isotype control and positive CD68 staining).

**Figure 2.**
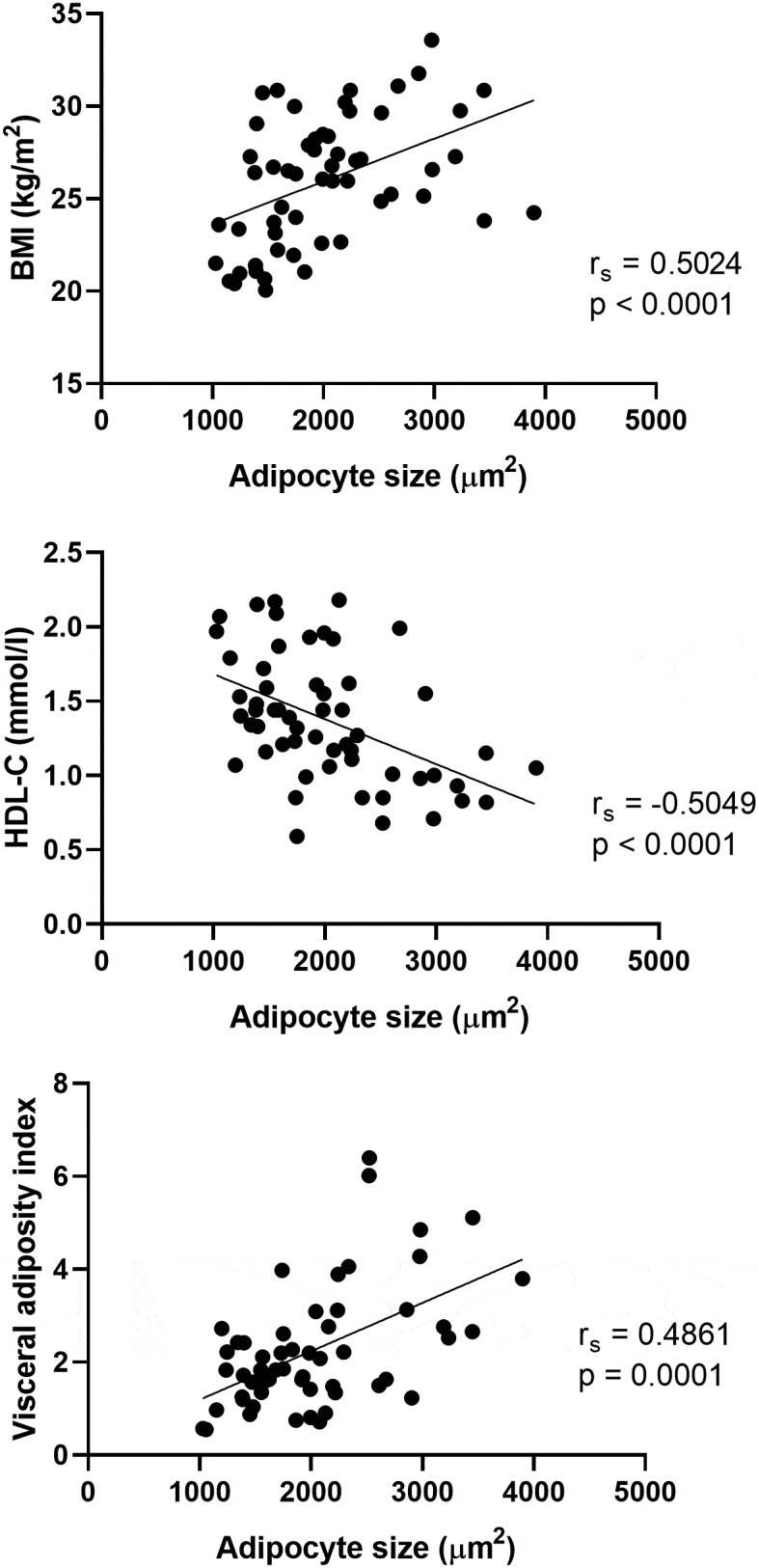
Adipocyte size correlates with various cardiovascular risk factors.

The average adipocyte area was 2020.34 ± 663.45 µm^2^. The differences in adipocyte size between men and women did not reach statistical significance, although the perivascular adipocytes tended to be bigger in men (data not shown). An intra-class correlation was used to analyse the agreement of two raters. The intra-class correlation coefficient was 0.953 for PVAT (*n* = 64). Representative images from adipocyte size analysis are shown in Figure 1(a,b). Data distribution is shown in Supplementary Figure S2. Adipocyte size correlated positively with many classical cardiovascular risk factors in PVAT from healthy LKDs. Selected graphs are shown in Figure 2. There was a strong positive relationship between perivascular adipocyte size and lipid metabolism parameters. We have found a positive correlation between adipocyte size on the one hand, and TG and remnant cholesterol levels on the other. Moreover, perivascular adipocyte size also correlated positively with overall body composition and insulin resistance (IR) as reflected by BMI, waist circumference, homoeostatic model assessment for insulin resistance index (HOMA-IR), glucose levels in plasma, and visceral adiposity index (VAI). In contrast, adipocyte size in PVAT correlated negatively with HDL-C levels and basal metabolic rate per kg (BMR/kg). All tested associations are shown in Table 2. Of those, correlations with waist circumference, BMI, VAI, TG, glucose, remnant cholesterol, and HDL-C levels remained significant even after false discovery rate (FDR) correction (Table 2).

r_s_ = Spearman’s rank correlation coefficient.

VAI – visceral adiposity index.

**Table 2.** Summary of correlation coefficients for adipocyte size in PVAT.

	PVAT (*N* = 56)	
	r_s_	p
Age (years)	0.1167	0.3915
Waist circumference (cm)	**0.3826**	**0.0036**
BMI (kg/m^2^)	**0.5024**	**<0.0001**
Body fat (%)	0.2114	0.1178
BMR (kcal/kg)	− 0.3141	0.0184
TG log10 (mmol/l)	**0.4107**	**0.0017**
HDL-C (mmol/l)	** − 0.5049**	**<0.0001**
LDL-C (mmol/l)	0.1035	0.4479
Remnant cholesterol (mmol/l)	**0.4063**	**0.0019**
hs-CRP (mg/l)	**0.3974**	**0.0052**
Glucose (mmol/l)	**0.4420**	**0.0009**
HOMA-IR	0.3104	0.0251
IL-1β (pg/ml)	0.1316	0.3888
TNF-α (pg/ml)	0.3318	0.0448
MCP-1 (pg/ml)	0.0672	0.6537
Adiponectin (pg/ml)	− 0.2138	0.1490
ICAM-1 (pg/ml)	0.2296	0.1205
VCAM-1 (pg/ml)	0.0546	0.7156
E-selectin (pg/ml)	0.3175	0.0296
Visceral adiposity index	**0.4861**	**0.0001**
CD68 + macrophages (%)	**0.4663**	**0.0019**
MAP-ATMs (%)	0.3047	0.0297
AI-ATMs (%)	− 0.3394	0.0226

Data in bold remained significant even after False discovery rate correction (two-stage linear step-up procedure of Benjamini, Krieger and Yekutieli).

Q: 1%.

Estimated number of true null hypotheses: 16.

Threshold: *p* values less than 0.0056 are ‘discoveries’.

r_s_ - Spearman’s rank correlation coefficient.

Surprisingly, no significant relationship was found between adipocyte size in PVAT and age, LDL-C levels, and body fat percentage. There was also no relationship between adipocyte size and plasma adiponectin levels.

### 2.2. Adipocyte size and its relationship to markers of inflammation

The proportion of anti-inflammatory adipose tissue macrophages (AI-ATMs) was slightly lower than the proportion of MAP-ATMs (26.13  ±  14.45% vs. 29.94  ±  13.81%). Although different transient subpopulations were also detected during the flow cytometric analysis, only the MAP-ATMs and AI-ATMs representing opposite ends of a phenotypical spectrum were chosen.

To confirm our results obtained by flow cytometry, adipose tissue samples were also stained immunohistochemically (Figure 1(c,d)). Interestingly, the proportion of CD68 + cells correlated with several cardiovascular risk factors. Of those, positive correlations with VAI and body fat percentage and a negative correlation with BMR remained significant even after correction (data not shown). Most notably, there was a strong positive correlation between the proportion of CD68 + cells and the MAP-ATM subpopulation.

**Figure 3.**
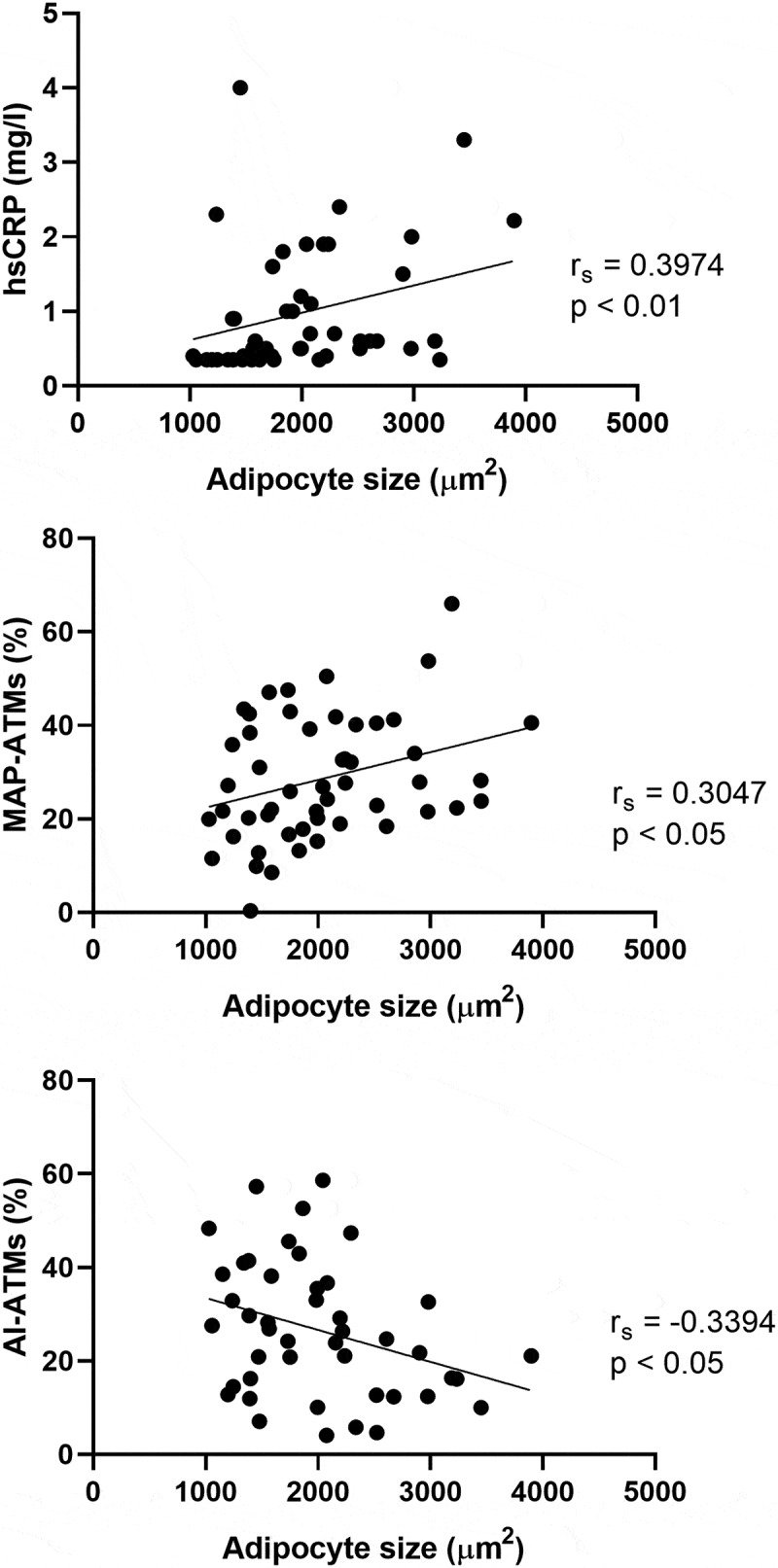
Adipocyte size correlates with markers of inflammation.

Adipocyte size in PVAT was associated with studied markers of inflammation – a positive correlation was found between the adipocyte size and the proportions of MAP-ATMs, hs-CRP, TNF-α and E-selectin levels in plasma and the percentage of CD68 + cells in adipose tissue. In contrast, the proportion of AI-ATMs correlated negatively with adipocyte size in PVAT. Only correlations with hs-CRP and the percentage of CD68 + cells remained significant after FDR correction. Selected graphs are shown in Figure 3.

r_s_ = Spearman’s rank correlation coefficient.

MAP-ATMs – metabolically active pro-inflammatory adipose tissue macrophages.

AI-ATMs – anti-inflammatory adipose tissue macrophages.

### 2.3. Principal component analysis confirms the correlation analyses and suggests a possible role of adhesion molecules

The principal component analysis (PCA) was then used to reduce the number of independent variables. First six components (linear combinations of the initial variables) explained 66% of the total variation. Tables with all eigenvalues (Supplementary Table S2) and eigenvectors (Supplementary Table S3) are shown in the Supplement. The first component, a linear combination of waist circumference, BMI, and VAI, explained 21% of the variability. The second component (body fat %, BMR/kg, TG, remnant cholesterol, VAI) explained 14% of the variability. The third component (adiponectin, ICAM-1, VCAM-1, E-selectin) explained 10% of the variability. The fourth component (LDL-C, MAP-ATMs, AI-ATMs) explained 8% of the variability. The fifth component (age, hs-CRP, HOMA-IR, E-selectin) explained 7% of the variability, and finally, the sixth component (IL-1β, MCP-1, percentage of CD68 + cells) explained 6% of the variability. The first component significantly correlated with adipocyte size (r_s_ = 0.6275, *p* < 0.0001). This confirms the results of correlation analyses. A significant correlation was also found for the third component representing mainly adhesion molecules (r_s_ = 0.4005, *p* = 0.0078).

## 3. Discussion

A large body of published data is focused on the relationships of adipocyte size to different cardiovascular risk factors in obese subjects. Thanks to the high number of kidney transplants from living donors in our institute, we were able to explore the close relationship between adipocyte size in a specific PVAT depot and cardiovascular risk factors in a cohort of healthy subjects.

The LKDs had similar or healthier lipid profile and BMI values compared to the general population sample. Only triglyceride levels were significantly higher in the LKDs group, together with a slight increase in plasma glucose, indicating that a long-term, lasting shift of criteria for living organ donors might enable the inclusion of a broader array of subjects (Table 1).

Adipocyte size in PVAT adjacent to the renal artery measured in the present study was comparable to adipocyte size previously measured in epicardial adipose tissue [26], adipose tissue surrounding the aorta, coronary artery and internal thoracic artery [27,28]. Adipocyte size in PVAT is usually (although not always [29]) reported to be significantly smaller in comparison to visceral and subcutaneous depots [26,28,30]. However, adipose tissue phenotype (gene expression, fibrosis, macrophage infiltration) can vary substantially even between different PVAT depots [27].

In our study, adipocyte size was related to lipid metabolism and body composition (Figure 2). There was a strong positive relationship between perivascular adipocyte size and markers of lipid metabolism (TG, HDL-C, remnant cholesterol). Moreover, perivascular adipocyte size correlated positively with parameters of body composition and glucose metabolism (BMI, waist circumference, glucose levels in plasma and VAI). Correlations of adipocyte size with BMI, waist circumference, TG, and HDL-C levels have been previously reported for visceral adipose tissue (VAT) and SAT [31–33]. Nevertheless, the relationship between BMI and adipocyte size has not been previously confirmed for epicardial adipose tissue (usually classified as PVAT in the literature due to its proximity to the coronary arteries) [26]. Interestingly, the relationship was shown to be less significant in the SAT depot of healthy subjects [34] or even completely lost in SAT of morbidly obese subjects (possibly due to significant hyperplasia) [35]. Moreover, the association between remnant cholesterol and adipocyte size is not explored in the current literature. Remnant cholesterol is an emerging risk factor for cardiovascular disease [36] and its link to adipose tissue dysfunction should stimulate further research. The association between adipocyte size and IR has been previously reported in omental and mesenteric VAT and SAT [11,30,31,37]. Our results, therefore, confirm the relationship between adipocyte size in PVAT and lipid metabolism and body composition. Most importantly, we were able to document these linear correlations in normal-weight healthy individuals.

Together with a hs-CRP measurement, flow cytometry was used to assess inflammation in adipose tissue. The relationship of two macrophage subtypes to adipocyte size was analysed (Figure 3). The positive correlations with MAP-ATMs and hs-CRP and the negative correlation with AI-ATMs suggest a link between both local and systemic inflammation and adipocyte size in PVAT. The observation of a potential relationship between the polarized macrophage subtypes and adipocyte size in PVAT in a healthy cohort of LKDs is a yet unreported finding and deserves further study.

Moreover, when analysed immunohistochemically (Figure 1(c,d)), the infiltration of CD68 + cells associated significantly with adipocyte size in PVAT. There is probably only a partial overlap of the CD68 + population and MAP-ATMs or AI-ATMs. The macrophage population in adipose tissue is phenotypically complex and changes dynamically during pathological stimuli [38]. It contains various intermediate subpopulations that may have remained undetected by our analyses. Moreover, similar to adipocyte size, macrophage infiltration differs significantly between various adipose tissue depots [26,28] and even between PVAT depots surrounding different vessels [27]. Data regarding macrophage infiltration in healthy adipose tissue are scarce and usually focused on SAT or VAT. Recently, a correlation between macrophage numbers in SAT and circulating inflammatory markers has been reported in healthy subjects [39]. The number of CD68 + cells correlated positively with adipocyte size and BMI in SAT obtained from children [40] and in SAT and omental fat in healthy adults [13,14]. Data regarding macrophage infiltration in PVAT come almost exclusively from patients with cardiovascular disease [10,41]. Our data, therefore, substantially expand current knowledge about early pre-atherosclerotic inflammatory changes in human PVAT.

The PCA confirmed the relationship between adipocyte size in PVAT and overall body composition (as reflected by the positive correlation with the first component). However, the significant positive correlation of adipocyte size with the third component (consisting of adiponectin, ICAM-1, VCAM-1 and E-selectin levels in plasma) was surprising because the simple correlations were not significant. Adiponectin production is known to be lower in obese subjects and correlates negatively with adipocyte size in SAT [42]. However, adiponectin production can differ in different adipose tissue depots [43]. Although the contribution of PVAT to serum adiponectin levels is probably minor, increased adiponectin levels were found in the PVAT adjacent to symptomatic carotid plaques [44]. The relationship between adipocyte size and adhesion molecule levels in plasma is not explored in the current literature. We can speculate, that their increase connected to an inflammatory state could further promote adipose tissue dysfunction and immune cell infiltration.

Our results are also in concordance with data from animal studies further outlining PVAT as a potential marker and therapeutic target in diabetes and atherosclerosis. PVAT was shown to be prone to inflammatory changes and adipocyte hypertrophy (induced by hypercaloric diet) in a prediabetic rat model [45]. Both high-fat and high-sucrose diets increased CD68 + cell infiltration in various PVAT depots [46]. Moreover, adipose tissue transplantation models have shown a strong influence of PVAT on atherosclerosis development in comparison to VAT and SAT. This was also associated with increased production of pro-inflammatory cytokines [47]. Inflammation and adipocyte hypertrophy in PVAT were reversed by dietary changes and sustained weight loss [48].

Our study has some limitations. Several methods are used routinely to measure adipocyte size [32]. The histological analysis used in this study underestimates cell size due to cell shrinkage caused by formalin fixation [32] and, possibly, also by subsequent incubation in hypertonic sucrose solution. Despite this limitation, results of different methods do correlate and associations with body fat parameters are not affected by the choice of measurement method [32,33]. The number of cells analysed histologically is also usually lower than with other methods; however, we decided to include a minimum of 400 adipocytes per sample to enhance the accuracy of our study (a representative analysed image is shown in Figure 1(a,b)). Moreover, a second blinded adipocyte size measurement was performed with high comparability further validating our results. Due to the cross-sectional design of our study, we were unable to determine whether the relationship between the presence of cardiovascular risk factors and adipocyte size was a causal one. It should also be noted that the associations between adipose tissue macrophage burden and cardiovascular risk factors (lipid parameters, markers of inflammation) have been recently shown to be at least partly confounded by adipocyte size in the SAT of moderately obese subjects [49], and similar confounding cannot be excluded in the PVAT depot in this study. Nevertheless, we were able to study clinically relevant properties of the rarely available PVAT from healthy human subjects and complement the results of animal studies [50].

In summary, our data show a strong association between the presence of cardiovascular risk factors on the one hand, and adipose tissue dysfunction and inflammation (represented by macrophage infiltration) on the other in a healthy cohort of living kidney donors. This confirms the recently established sensory role of adipose tissue in atherosclerosis [51]. It is particularly the perivascular depot which is sensitive to early metabolic and inflammatory changes before the onset of atherosclerosis, further supporting the use of adipose tissue inflammation as a suitable marker in primary prevention.

## 4. Patients/methods/materials

### 4.1. Study subjects

Eighty-three LKDs were enrolled in the study performed at the Institute for Clinical and Experimental Medicine in Prague, Czech Republic. Analysis of a similar group of LKDs was published earlier [52] without determining adipocyte size. All participants signed an informed consent form and completed a standardized questionnaire with a study nurse to identify their cardiovascular risk factors and medication history. At least one sample PVAT was available in 69 LKDs. Their detailed characteristics and comparison with controls from the general population [25] are shown in Table 1. The design of the present study was approved by the joint Ethics Committee of the Institute for Clinical and Experimental Medicine and Thomayer University Hospital, Prague, Czech Republic (approval reference number: G-16-06-22) and complied with the ethical principles of the Declaration of Helsinki (2013). The data that support the findings of this study are openly available in Open Science Framework at http://doi.org/10.17605/OSF.IO/QW76A [53].

### 4.2. Clinical and biochemical data

Fasting blood samples were collected preoperatively immediately before anaesthesia induction. Plasma total cholesterol, HDL-C, and TG concentrations were determined enzymatically on a Cobas Mira Plus Autoanalyzer using commercially available kits (Roche Diagnostics). The concentration of low-density lipoprotein-cholesterol (LDL-C) [54] and remnant cholesterol [55] was calculated. High-sensitivity C-reactive protein (hsCRP) and insulin concentrations were measured in plasma with commercially available kits using immunoturbidimetric and immunoradiometric assays (Roche Diagnostics; Beckman Coulter), respectively. Glucose concentrations were obtained from recent medical records and the HOMA-IR was calculated. Based on waist circumference, BMI, and lipid parameters, the VAI was calculated as proposed by Amato et al. [56].

The plasma concentrations of selected cytokines, specifically IL-1β, TNF-α, MCP-1, adiponectin, ICAM-1, VCAM-1, and E-selectin, were measured by ProcartaPlex Immunoassays (Thermo Fisher Scientific). Plates were read using a LABScan3D (One Lambda) and xPonent software (Thermo Fisher Scientific). Body fat percentage and basal metabolic rate per kg (BMR/kg) were assessed by bioelectrical impedance analysis using a Bodystat 1500 system (Bodystat Limited) one day prior to surgery. Impedance was measured in the supine position with arms and legs apart and body composition (e.g. body fat percentage) and BMR/kg were calculated using predefined predictive equations.

### 4.3. Adipose tissue samples

Samples of PVAT adjacent to the renal artery were obtained during hand-assisted retroperitoneoscopic nephrectomy and immediately transported to laboratory. Small pieces (approximately 5–10 mm) of adipose tissue were excised and fixed overnight at room temperature in neutral buffered formalin (Diapath). After washing with DPBS (Biosera), the tissue was incubated overnight in DPBS sucrose buffer (30% solution) at 4°C (PENTA). Samples were then gently dried with cellulose wadding paper, mounted in Tissue-Tek O. C. T.™ Compound (Sakura Finetek) and flash frozen in liquid nitrogen using isopentane bath (Carl Roth) and stored at  −80°C until further use.

Freshly prepared cryosections (8 μm) were incubated in 96% ethanol (5 minutes, PENTA). After short wash in distilled water, the sections were stained with fresh Giemsa working solution (10 minutes) (PENTA), washed thoroughly in tap water and dried at room temperature. Dry sections were shortly cleared in xylene (Carl Roth) and immediately mounted (Pertex®, Histolab AB). Four representative photographs of each section were taken at 10 × magnification using the Eclipse Ni-E upright microscope (Nikon Europe B. V.) to measure adipocyte area and diameter (Fiji Adiposoft plugin, a semi-automatic approach). Damaged adipocytes and adipocytes on image borders were excluded from the analysis. A minimum of 400 cells was analysed per sample. Due to our strict criteria regarding the minimal number of measured adipocytes, small amount of tissue available or poor quality of histological sections, 13 PVAT samples were excluded from the study. Adipocyte size was expressed as an area in μm^2^.

The presence of CD68 + cells was confirmed immunohistochemically. Cryosections were fixed in an ice-cold acetone-methanol mixture (1:1, PENTA) for 10 minutes. Heat-induced epitope retrieval was performed with Antigen Unmasking Solution (Tris-Based, 25 minutes, 95°C, Vector Laboratories). After washing, sections were blocked for 2 hours in DPBS with goat serum (5%), bovine serum albumin (1%) (both Biosera) and Triton X (0.3%, Sigma Aldrich) and incubated with primary antibody (CD68; 1:100; clone PG-M1, Agilent Technologies) or isotype control (Biolegend) overnight. After 1-hour incubation with a secondary antibody (goat anti-mouse antibody DyLight 488; Thermo Fisher Scientific), sections were mounted in DAPI mounting medium (Abcam). Four representative photographs of each section were taken at 10 × magnification using the Eclipse Ni-E upright microscope. The number of CD68 + cells was expressed as the percentage of all nuclei present. Only samples with present CD68 + cells were included in correlation analyses. Representative histological and immunohistochemical images are shown in Figure 1.

Part of the adipose tissue samples was processed for flow cytometry. After removal of visible blood vessels and connective tissue, adipose tissue was cut into smaller pieces and digested in collagenase II solution (2 mg/ml, 3.4 ml per g of tissue, Sigma Aldrich) for 20 minutes at 37°C in shaking water bath. The resultant cell suspension was cooled, repeatedly filtered on ice (150 μm and 50 μm filters, Sysmex) and centrifuged (10 minutes, 200 rcf, 4°C) twice to obtain the stromal vascular fraction. Based on our previous results, the proportions of macrophage subpopulations were studied in isolated stromal vascular fractions based on the expression of the CD14, CD16, CD36 and CD163 markers (CD14 – PC7, clone RMO52; CD16 – ECD, clone 3G8; CD36 – FITC, clone FA6.152 (all Beckman Coulter); eBioscience Fixable Viability Dye – eFluor 780 (FVD, Thermo Fisher Scientific); CD163 – PE, clone RM3/1 (Biolegend). Purified cell suspensions from all tissues were incubated with a mix of conjugated antibodies for 20 minutes at room temperature and subsequently analysed by flow cytometry (CytoFLEX, Beckman Coulter). The number of live cells was determined by the absence of FVD staining; only singlets were included in the analysis. The threshold for CD16 positivity was set on a blood sample obtained from the same patient. Data were analysed using FlowJo software (Becton, Dickinson & Company) Of those, the pro-inflammatory MAP-ATMs subpopulation (CD14 + CD16 + CD36^high^) was analysed further. The detailed flow cytometric data were published previously [52]. Moreover, the anti-inflammatory adipose tissue macrophage subpopulation (AI-ATMs, CD14 + CD16-CD163 + ) was studied in the present study. The gating strategy is shown in Supplementary Figure S1. Catalogue numbers of used reagents are listed in the Supplementary Table S1.

### 4.4. Statistical analysis

Data were analysed using JMP 16.2.0, 2020–2021 (SAS Institute Inc.) and GraphPad Software, and their distribution was checked using the D’Agostino & Pearson normality test. Missing data were not approximated. An intra-class correlation method was used to evaluate the inter-observer variability and for data validation (data not shown). Differences in adipocyte size between sexes were compared using the Mann-Whitney test. An unpaired t-test was used to compare basic lipid parameters in LKDs and an age and sex-matched control group from the MONICA study (data with non-normal distribution were log-transformed). Spearman´s correlation was used to identify potential associations between the variables. The false discovery rate (FDR) approach was used to correct the multiple comparisons and refine the results of our analysis with the FDR control using the two-stage linear step-up procedure proposed by Benjamini, Krieger and Yekutieli (FDR = 1%). To decrease the dimensionality of the data set, the principal components analysis method was used for the selected variables from Table 2 (*N* = 23). All input variables were standardized. Spearman´s correlation was then used to identify potential associations between the six principal components and adipocyte size. A second adipocyte size measurement was performed by a blinded researcher to validate our data. Intra-class correlation was used to analyse the agreement of two raters. All measured samples (including those with less than 400 measured cells per sample) were used for validation analysis. A two-way model with same raters for all subjects was chosen. Only data from the first measurement are presented in the Results section. The two-sided significance level was set at 0.05.

## Supplementary Material

Supplemental Material

## Data Availability

The data that support the findings of this study are openly available in Open Science Framework at http://doi.org/10.17605/OSF.IO/QW76A, reference number [53].

## Abbreviations

AI-ATMs: anti-inflammatory adipose tissue macrophages
BMI: body mass index
BMR/kg: basal metabolic rate per kg
HDL-C: high-density lipoprotein cholesterol
hs-CRP: high-sensitivity C-reactive protein
HOMA-IR: homoeostatic model assessment for insulin resistance index
ICAM-1: intercellular adhesion molecule 1
IL-1β: interleukin-1β
IR: insulin resistance
LDL-C: low-density lipoprotein cholesterol
LKDs: living kidney donors
MAP-ATMs: metabolically active pro-inflammatory adipose tissue macrophages
MCP-1: monocyte chemoattractant protein 1
PCA: principal component analysis
PVAT: perivascular adipose tissue
SAT: subcutaneous adipose tissue
TG: triglycerides
VAI: visceral adiposity index
VAT: visceral adipose tissue
VCAM-1: vascular cell adhesion molecule 1
TNF-α: tumour necrosis factor α
