# Supplementary material for: Perivascular adipocyte size is related to the lipid profile and inflammatory changes in a healthy population
Source: Adipocyte. 2025 May 23;14(1):2499500. doi: 10.1080/21623945.2025.2499500 (PMC12118406; doi:10.1080/21623945.2025.2499500)
Supplement: Supplemental Material [file KADI_A_2499500_SM7945.zip › Alt text for Supplementary material.docx]

**Alt text for Supplementary material**

**Supplementary figure 1 alt text**

The gating process shown in nine subsequent pseudocolor dot plot graphs with outlined gates. The first six graphs show the process for a representative adipose tissue sample, and the last three graphs show how the gate thresholds were set on a blood sample from the same subject.

**Supplementary figure 2 alt text**

A) Bar graph showing the lognormal distribution of adipocyte size in perivascular adipose tissue with higher bars on the left.

B) Bar graph showing normal distribution of adipocyte size in the perivascular adipose tissue after logarithmic transformation.
