# Supplementary material for: Perivascular adipocyte size is related to the lipid profile and inflammatory changes in a healthy population
Source: Adipocyte. 2025 May 23;14(1):2499500. doi: 10.1080/21623945.2025.2499500 (PMC12118406; doi:10.1080/21623945.2025.2499500)
Supplement: Supplemental Material [file KADI_A_2499500_SM7945.zip › Supplement_Adip_stat_corr_final_rpc_3_CLEAN doc.docx]

**Supplementary Material**

**Perivascular adipocyte size is related to the lipid profile and inflammatory changes in a healthy population**

Hana Bartuskova^1,2^*, Ivana Kralova Lesna^1,3^, Sona Kauerova^1^, Vera Lanska^4^, Jiri Fronek^5^, Libor Janousek^5^, Barbora Muffova^1,2^, Karel Paukner^1,2^, Rudolf Poledne^1^

^1^Atherosclerosis Research Laboratory, Experimental Medicine Center, Institute for Clinical and Experimental Medicine, Prague, Czech Republic;

^2^Department of Physiology, Faculty of Science, Charles University, Prague, Czech Republic;

^3^Department of Anaesthesiology, Resuscitation and Intensive Care Medicine, 1^st^ Faculty of Medicine of Charles University and Military University Hospital, Prague, Czech Republic;

^4^Department of Data Science and Statistics, Information Technology Division, Institute for Clinical and Experimental Medicine, Prague, Czech Republic;

^5^Transplantation Surgery Department, Transplantation Center, Institute for Clinical and Experimental Medicine, Prague, Czech Republic

**Table of content:**

1. Supplementary Figure 1: Gating strategy
2. Supplementary Figure 2: Adipocyte size distribution testing
3. Supplementary Table 1: Materials and reagentsSupplementary Table 2: Eigenvalues
4. Supplementary Table 3: Eigenvectors

Supplementary Figure 1: Gating strategy


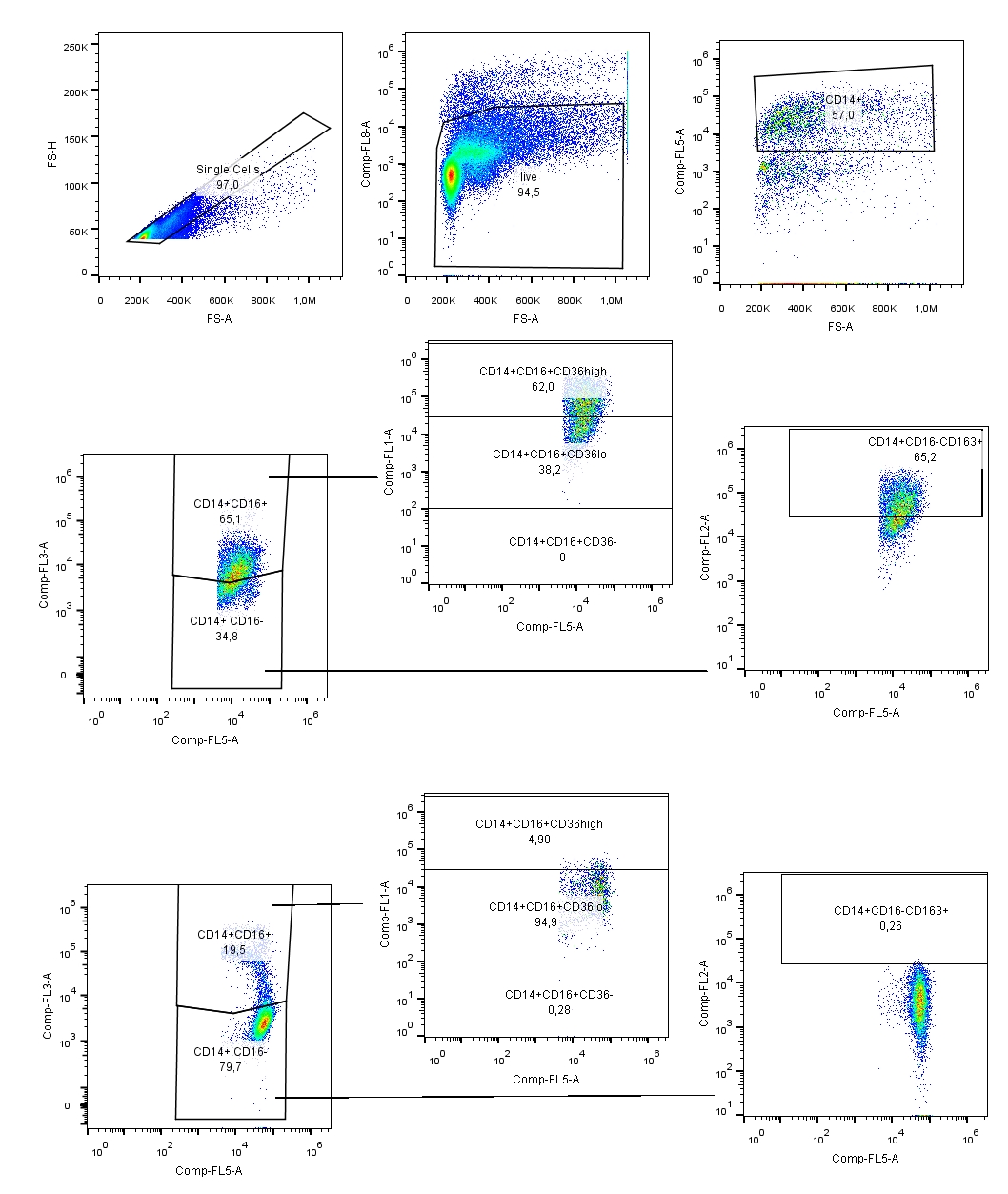


After the exclusion of doublets and death cells (FVD positive), only CD14+ cells were further analyzed for the presence of the CD16 marker. The threshold for CD16 positivity was set on a blood sample from the same patient (bottom line). Expression of CD36 marker was assessed in CD16+ cells. Expression of CD163 marker was assessed in CD16- cells.

Supplementary Figure 2: Adipocyte size distribution testing

A)

**Fitted Normal Distribution**

**Goodness-of-Fit Test**

|  | **W** | **Prob<W** |
| --- | --- | --- |
| Shapiro-Wilk | 0.940245 | 0.0080* |

Note: Ho = The data is from the Normal distribution. Small p-values reject Ho.

**Fitted Lognormal Distribution**

**Goodness-of-Fit Test**

|  | **A˛** | **Simulated p-Value** |
| --- | --- | --- |
| Anderson-Darling | 0.2288339 | 0.8200 |

Note: Ho = The data is from the Lognormal distribution. Small p-values reject Ho.

**B)**

**Fitted Normal Distribution**

**Goodness-of-Fit Test**

|  | **W** | **Prob<W** |
| --- | --- | --- |
| Shapiro-Wilk | 0.9747549 | 0.3446 |

Note: Ho = The data is from the Normal distribution. Small p-values reject Ho.

The adipocyte size data distribution was lognormal (A). After logarithmic transformation, the normal distribution is not rejected (B).

Supplementary Table 1: Materials and Reagents.

| **Description** | **Vendor** | **Cat. number** |
| --- | --- | --- |
| CD14-PC7 | Beckman Coulter | A22331 |
| CD16-ECD | Beckman Coulter | B49216 |
| CD36-FITC | Beckman Coulter | B49201 |
| CD163-PE | Biolegend | 326506 |
| Bovine serum albumin | Biosera | PM-T1727 |
| Collagenase from Clostridium histolyticum | Sigma-Aldrich | C6885 |
| Dulbecco's phosphate buffered saline | Biosera | LM-S2041 |
| eBioscience™ Fixable Viability Dye eFluor™ 780 | Thermo Fisher Scientific | 65-0865-14 |
| Tissue-Tek O. C. T ^™^ Compound | Sakura Finetek | 4583 |
| Giemsa-Romanowski solution | PENTA | 14460-11000 |
| Pertex® mounting medium | Histolab | HI-00801 |
| Antigen Unmasking Solution, Tris-Based | Vector Laboratories | H-3301-250 |
| Goat Serum | Biosera | GO-605/100 |
| Triton^™^ X-100 | Sigma-Aldrich | X100-5ML |
| CD68 antibody (mouse) | Agilent Technologies | M0876 |
| Purified Mouse IgG3, κ Isotype Ctrl Antibody | BioLegend | 401301 |
| Goat anti-Mouse IgG (H+L) Cross-Adsorbed Secondary Antibody, DyLight 488 | Invitrogen | 35503 |
| Mounting Medium With DAPI | Abcam | ab104139 |
| Insulin(e) IRMA kit | Beckman Coulter | IM3210 |
| ProcartaPlex Human Basic Kit | Invitrogen | EPX010-10420-901 |
| ProcartaPlex Human Simplexes | Invitrogen | Il-1β: EPX01A-10224-901  TNF-α: EPX01A-10223-901  MCP-1: EPX01B-10281-901  Adiponectin: EPX01A-12032-901  ICAM-1: EPX01A-10201-901  VCAM-1: EPX01A-10232-901  E-selectin: EPX01A-10205-901 |

Supplementary Table 2: Eigenvalues

| **Number** | **Eigenvalue** | **Percent** |  | **Cum Percent** |
| --- | --- | --- | --- | --- |
| 1 | 4.780963 | 20.787 |  | 20.787 |
| 2 | 3.169443 | 13.780 |  | 34.567 |
| 3 | 2.450959 | 10.656 |  | 45.223 |
| 4 | 1.888359 | 8.210 |  | 53.434 |
| 5 | 1.547479 | 6.728 |  | 60.162 |
| 6 | 1.365706 | 5.938 |  | 66.100 |
| 7 | 1.319188 | 5.736 |  | 71.835 |
| 8 | 1.040307 | 4.523 |  | 76.358 |
| 9 | 0.932333 | 4.054 |  | 80.412 |
| 10 | 0.762693 | 3.316 |  | 83.728 |
| 11 | 0.669971 | 2.913 |  | 86.641 |
| 12 | 0.631495 | 2.746 |  | 89.386 |
| 13 | 0.549062 | 2.387 |  | 91.774 |
| 14 | 0.505488 | 2.198 |  | 93.972 |
| 15 | 0.399165 | 1.735 |  | 95.707 |
| 16 | 0.311502 | 1.354 |  | 97.061 |
| 17 | 0.256425 | 1.115 |  | 98.176 |
| 18 | 0.138090 | 0.600 |  | 98.777 |
| 19 | 0.112602 | 0.490 |  | 99.266 |
| 20 | 0.087961 | 0.382 |  | 99.649 |
| 21 | 0.040484 | 0.176 |  | 99.825 |
| 22 | 0.032265 | 0.140 |  | 99.965 |
| 23 | 0.008060 | 0.035 |  | 100.000 |

Eigenvalues represent the percentage of explained variance (column cum percent). First six components explain 66% of total variation.

Supplementary Table 3: Eigenvectors

|  | **Prin1** | **Prin2** | **Prin3** | **Prin4** | **Prin5** | **Prin6** |
| --- | --- | --- | --- | --- | --- | --- |
| Age | 0.11074 | 0.17446 | 0.02990 | -0.06601 | 0.41676 | -0.06588 |
| Waist circumference | 0.31877 | 0.05273 | -0.09351 | 0.10911 | -0.14161 | -0.19843 |
| BMI | 0.33717 | 0.15831 | -0.03371 | 0.09138 | -0.16676 | -0.26076 |
| Body fat % | 0.22296 | 0.34556 | -0.09708 | 0.12107 | 0.23505 | -0.20220 |
| BMR/kg | -0.29230 | -0.31317 | 0.07041 | -0.14695 | -0.19480 | 0.29508 |
| TG (log10) | 0.28719 | -0.35854 | -0.00611 | -0.04341 | 0.11888 | -0.13656 |
| HDL-C | -0.22494 | 0.22712 | -0.15919 | -0.10432 | 0.02247 | -0.00163 |
| LDL-C | 0.13044 | 0.19340 | -0.03771 | -0.38039 | 0.05144 | -0.10608 |
| Remnant cholesterol | 0.28101 | -0.39337 | 0.02602 | 0.00943 | 0.08702 | -0.06430 |
| Hs-CRP | 0.18724 | 0.23068 | 0.13669 | -0.22590 | -0.33971 | 0.05847 |
| GLucose | 0.18755 | 0.07049 | 0.13342 | 0.08431 | 0.04777 | 0.19302 |
| HOMA-IR | 0.20639 | 0.22132 | 0.20518 | -0.29684 | -0.30492 | 0.14345 |
| IL-1β | 0.17297 | 0.08662 | -0.18356 | 0.22492 | -0.23061 | 0.36360 |
| TNF-α | 0.09970 | -0.13580 | -0.03248 | 0.19331 | -0.17562 | 0.08811 |
| MCP-1 | 0.24350 | 0.05123 | 0.01120 | 0.26457 | 0.02859 | 0.45299 |
| Adiponectin | -0.08966 | 0.23294 | 0.30033 | 0.18380 | 0.20900 | 0.23963 |
| ICAM-1 | -0.05268 | 0.00074 | 0.53896 | 0.19527 | 0.04910 | -0.18275 |
| VCAM-1 | -0.09383 | 0.03974 | 0.53503 | 0.20536 | 0.09764 | -0.07931 |
| E-selectin | 0.14757 | -0.00637 | 0.33700 | -0.04672 | -0.35566 | -0.03456 |
| Visceral adiposity index | 0.30157 | -0.35376 | 0.04721 | 0.08873 | 0.10726 | -0.00270 |
| CD68 | 0.19042 | 0.02805 | -0.01377 | -0.08366 | 0.30193 | 0.42441 |
| MAP-ATMs | 0.15597 | 0.01822 | 0.09887 | -0.37859 | 0.27480 | 0.20345 |
| AI-ATMs | -0.05761 | 0.18458 | -0.20300 | 0.45046 | -0.06782 | 0.00681 |

Components (Prin1 – Prin6) in PCA are a linear combination of variables. Eigenvectors are coefficients defining the linear combination. Only those higher than 0.3 were included (highlighted in yellow).
